# Compliance with the Prescription of Antihypertensive Medications and Blood Pressure Control in Primary Care

**DOI:** 10.5935/abc.20170009

**Published:** 2017-02

**Authors:** Mayra Faria Novello, Maria Luiza Garcia Rosa, Ranier Tagarro Ferreira, Icaro Gusmão Nunes, Antonio José Lagoeiro Jorge, Dayse Mary da Silva Correia, Wolney de Andrade Martins, Evandro Tinoco Mesquita

**Affiliations:** 1 Departamento de Medicina Clínica - Universidade Federal Fluminense, Niterói, RJ - Brazil; 2 Departamento de Epidemiologia e Bioestatística - Universidade Federal Fluminense, Niterói, RJ - Brazil; 3 Departamento de Fundamentos de Enfermagem e Administração - Universidade Federal Fluminense, Niterói, RJ - Brazil

**Keywords:** Hypertension, Arterial Pressure, Control, Drug Prescription, Antihypertensive Agents

## Abstract

**Background:**

Hypertension is the most prevalent risk factor for cardiovascular disease, and its proper control can prevent the high morbidity and mortality associated with this disease.

**Objective:**

To assess the degree of compliance of antihypertensive prescriptions with the VI Brazilian Guidelines on Hypertension and the blood pressure control rate in primary care.

**Methods:**

Cross-sectional study conducted between August 2011 and November 2012, including 332 adults ≥ 45 years registered in the Family Doctor Program in Niteroi and selected randomly. The analysis included the prescribed antihypertensive classes, doses, and frequencies, as well as the blood pressure (BP) of the individuals.

**Results:**

The rate of prescription compliance was 80%. Diuretics were the most prescribed medications, and dual therapy was the most used treatment. The most common non-compliances were underdosing and underfrequencies. The BP goal in all cases was < 140/90 mmHg, except for diabetic patients, in whom the goal was set at < 130/80 mmHg. Control rates according to these goals were 44.9% and 38.6%, respectively. There was no correlation between prescription compliance and BP control.

**Conclusions:**

The degree of compliance was considered satisfactory. The achievement of the targets was consistent with national and international studies, suggesting that the family health model is effective in BP management, although it still needs improvement.

## Introduction

Hypertension is highly prevalent in Western populations, easily identifiable, and susceptible of treatment, comprising one of the most important risk factors for cardiovascular disease.^1^ The prevalence of hypertension has been increasing along with the increase in life expectancy and changes in lifestyle, with emphasis on the increase of overweight and obesity.^1^ According to estimates, one-third of the world population will be hypertensive in 2025, a fact that will inexorably bring serious consequences to global public health, particularly through damage in target organs.^1,2^

In spite of solid scientific evidence showing benefits of antihypertensive treatment in the reduction of cardiovascular risk,^3,4^ control of blood pressure (BP) to values < 140/90 mmHg is achieved in less than one-quarter of the individuals diagnosed with hypertension and in one-third of those receiving treatment for hypertension, even in countries with a well-structured health system.^5-7^ In Brazil, the rates of effective BP control are between 10% and 57%.^8^ Health system deficiencies, low adherence to treatment by the patients, and inertia to start and intensify the therapy and achieve therapeutic goals contribute to the ineffectiveness in controlling this disease.^6,9^

In hypertensive patients, BP control within the recommendations of the guidelines is one of the ways to evaluate the quality of care in hypertension.^10-12^ Although most physicians consider the hypertension guidelines as valid, the degree of compliance with the recommendations varies widely, with low implementation of the recommendations in clinical practice resulting in impaired quality of care.^13-17^ The compliance of the prescriptions of antihypertensive drugs to current recommendations has also been used as a way of assessing the quality of care.^18^

The aim of this study was to assess the quality of care of hypertensive patients aged 45 to 99 years enrolled in the Family Doctor Program (*Programa Médico de Família*, PMF) in Niterói, Rio de Janeiro, through the assessment of compliance of the prescription of antihypertensive drugs to the VI Brazilian Guidelines for Hypertension (*VI Diretrizes Brasileiras de Hipertensão*, VI DBH) and the rate of BP control.

## Methods

The present study is part of the Digitalis Study, a cross-sectional study conducted between August 2011 and November 2012, including a random sample of the population registered in the PMF in Niterói (state of Rio de Janeiro, Brazil), of both sexes, aged between 45 and 99 years. Details of the methodology of the Digitalis Study have been previously published.^19^

The individuals were invited to attend the PMF unity close to their homes. The researchers were trained and tested in a pilot study carried out in one of the units not included in the analysis.

All patients were evaluated with medical history, complete physical examination (including anthropometry), and complementary tests. The BP was determined with the patient in the sitting position, using a digital sphygmomanometer (Omron 711 HC, Omron, Kyoto, Japan),^20^ with three measurements with an interval of 1 minute between each measurement. In the event of a difference greater than 5 mmHg, a fourth measurement was obtained. The mean BP was calculated considering all the measurements obtained. The precautions taken before and during the BP assessment followed the recommended by the Brazilian Society of Cardiology.^21^

The evaluations performed in this study included the level of physical activity of the individuals, classifying them according to the International Physical Activity Questionnaire (IPAQ) into four items: sedentary, irregularly active, active, and very active.^22^ The individuals who did not perform any physical activity for at least 10 continuous minutes during the week in which the questionnaire was applied were considered to be sedentary. To evaluate the consumption of alcohol, we considered a consumption of risk when the daily average exceeded two doses for males and one dose for females.^23^

The inclusion criteria comprised declared hypertension and/or use of antihypertensive pharmacological treatment, and ability to provide information about medications in use (prescription and/or medication package and/or complete verbal information about the prescription).

The goals recommended by the VI DBH^21^ were adopted and BP < 140/90 mmHg was defined as the cut-off point for therapeutic efficacy. An exception was made for diabetic patients, whose goal was defined as BP < 130/80 mmHg. The indicators of prescription compliance were defined after revision of the VI DBH,^21^ establishing the classes of medications that should ideally be prescribed (alone or in combination), as well as the recommended doses and frequencies. In the case of monotherapy, we considered as compliant those prescriptions including the following classes: angiotensin-converting enzyme inhibitors (ACEi); angiotensin receptor blockers (ARB); adrenergic beta-blockers; thiazide, loop, and potassium-sparing diuretics; or calcium channel blockers. In cases of associations, we evaluated whether the associations were known to be effective and if there were associations of drugs of the same class, with the exception of loop or thiazide diuretics with spironolactone. In the case of triple therapy, we observed whether the prescription included one diuretic, as recommended by the VI DBH.^21^ We considered as "underdoses" those doses prescribed below the minimum recommended dose by the VI DBH^21^ and as "underfrequencies" those prescriptions containing medications prescribed in a frequency of administration below the recommended minimum. Opposite criteria were used to define the "overdoses" and "overfrequencies". A table with all doses and frequencies recommended by the VI DBH was used to classify the prescriptions.

The analysis of the prescriptions was initially performed considering only the drugs prescribed, without considering the effectiveness of the treatment (BP control). According to this analysis, the prescriptions were categorized as "compliant" or "non-compliant".

A consolidated analysis considering both the prescription compliance and the adequacy of the BP control categorized patients as being "treatment compliant" when having a compliant prescription associated with controlled BP.

### Statistical analysis

The characteristics of the individuals and the prescriptions are presented in absolute and relative frequencies. We used Pearson's chi-square test for comparison between groups. The level of statistical significance was set at 5%. All analyses were performed using the statistical package SPSS, version 21 (IBM Corp., Armonk, NY, USA).

### Ethical considerations

This study was conducted according to the principles set in the Resolution 466/2012 of the National Commission for Research Ethics (CONEP). The study protocol was submitted to the Committee for Ethics in Research of the Hospital Antônio Pedro and approved at the plenary meeting of June 11, 2010, under the CAAE number: 0077.0.258.000-10. All subjects signed an informed consent form.

## Results

The Digitalis Study evaluated 633 individuals, of whom 332 (53%) were included in the present analysis after meeting the inclusion criteria. Table 1 presents the demographic and clinical characteristics of the study patients. The prescribed medications were analyzed in most cases through direct verification of the prescriptions (56.4%), followed by precise information given by the patient or family member (34.3%), and from the medication packaging presented by the patient or family member (9.3%). Table 2 shows the main prescribed medications according to their classes. Table 3 presents the prescription types (monotherapy or association).

**Table 1:** Characteristics of the patients

Characteristics	Individuals analyzed n (%)
**Sex**	
Male	103 (31.0)
Female	229 (69.0)
**Age (years)**	
45 - 59	167 (50.3)
60 - 69	90 (27.1)
≥ 70	75 (22.6)
**Self-declared color**^$^	
White	108 (32.7)
Hybrid (Black/White)	126 (38.2)
Black	96 (29.1)
**Smoking**	
Never smoked	168 (50.6)
Ex-smoker	108 (32.5)
Smoker	56 (16.9)
**Excessive intake of alcohol**	
Yes	24 (7.2)
No	308 (92.8)
**Physical activity**^&^	
Sedentary	77 (27.9)
Irregular	40 (14.5)
Active	140 (50.7)
Very active	19 (6.9)
**Obesity**^#^	
BMI < 26 (ideal weight)	106 (32.1)
BMI 26 - 29.99 (overweight)	104 (31.5)
BMI ≥ 30	120 (36.4)
**Diabetes**	
Yes	107 (32.2)
No	225 (67.8)

BMI: body mass index.

$ The color was not informed by two individuals.

& The level of physical activity was not verified in 56 individuals.

# The BMI (kg/m2) was not evaluated in two individuals.

**Table 2:** Main prescribed medications (n=332)

Name	N (%)
**Diuretics**	209 (63.0)
Hydrochlorothiazide	178 (53.7)
Furosemide	16 (4.8)
Chlorthalidone	10 (3.0)
Spironolactone	3 (0.9)
Indapamide	2 (0.6)
**ACEi**	192 (57.8)
Enalapril	108 (32.5)
Captopril	81 (24.4)
Lisinopril	1 (0.3)
Ramipril	1 (0.3)
Cilazapril	1 (0.3)
**BB**	89 (26.8)
Atenolol	53 (16.0)
Propranolol	27 (8.1)
Carvedilol	7 (2.1)
Metoprolol	1 (0.3)
Bisoprolol	1 (0.3)
**CCB**	84 (25.3)
Amlodipine	41 (12.3)
Nifedipine	37 (11.2)
Diltiazem	4 (1.2)
Verapamil	1 (0.3)
Lercanidipine	1 (0.3)
**ARB**	61 (18.4)
Losartan	52 (15.7)
Valsartan	9 (2.7)
**Others**	14 (4.2)
Clonidine	8 (2.4)
Hydralazine	4 (1.2)
Aliskiren	1 (0.3)
Methyldopa	1 (0.3)

ACEi: angiotensin-converting enzyme inhibitors; BB: beta-blockers; CCB: calcium channel blockers; ARB: angiotensin receptor blockers.

**Table 3:** Main associations of medications (n=332)

Type of therapy	Prescriptions N (%)	Diuretics N (%)	ACEi N (%)	BB N (%)	CCB N (%)	ARB N (%)
Monotherapy	115 (34.6)	25 (21.7)	61 (53.0)	8 (7.0)	7 (6.1)	14 (12.2)
Dual therapy	136 (41.0)	112 (41.6)	85 (31.6)	30 (11.2)	24 (8.9)	18 (6.7)
Triple therapy	60 (18.1)	50 (28.8)	34 (19.5)	34 (19.5)	35 (20.1)	21 (12.1)
> 3 medications	21 (6.3)	21 (27.6)	12 (15.8)	17 (22.4)	18 (23.7)	8 (10.5)

ACEi: angiotensin-converting enzyme inhibitors; BB: beta-blockers; CCB: calcium channel blockers; ARB: angiotensin receptor blockers.

In 80.72% of the cases (268 prescriptions), the prescriptions were compliant with the VI DBH^21^ with respect to the medication chosen and their dosages and frequencies prescribed by the family doctor.

Table 4 shows the main non-compliances encountered. Of note, only 1.8% (six prescriptions) presented more than one simultaneous non-compliance. We observed that the non-compliances relative to underdosing and underfrequencies of medications were the ones most commonly found. The use of losartan potassium twice daily, prescribed in approximately 30% of the cases, was not considered a non-compliance despite a recommendation by the VI DBH of a single daily dose, since the prescription of two daily doses has pharmacological support^24^ and is recommended by other guidelines.

**Table 4:** Main non-compliances encountered

Prescribed Medication	Total	Underdosing	Overdosing	Underfrequency	Overfrequency	Triple therapy without diuretic	Wrong association
	N (%)	N (%) medication	N (%) medication	N (%) medication	N (%) medication	N (%)	N (%) medication
Diuretics	11 (16)	0	0	0	2 (18) HCTZ	8 (73)	1 (9) HCTZ + Chlorthalidone
ACEi	20 (29)	1 (5) Enalapril	0	10 (50) Captopril	3 (15) Enalapril	-	6 (30) ACEi + ARB
BB	15 (21.7)	3 (20) PPL	0	3 (20) Carvedilol 7 (47) PPL	2 (13) Atenolol	-	-
CCB	18 (26.1)	3 (16.7) Nifedipine 3 (16.7) Diltiazem	4 (22.2) Amlodipine	8 (44.4) Nifedipine	0	-	-
ARB	0	0	0	0	0	-	ARB + ACEi #
Other (Clonidine / Hydralazine / Methyldopa)	5 (7.2)	2 (40) Hydralazine	0	2 (40) Hydralazine	0	-	1 (20) Clonidine + HCTZ
TOTAL	69 (100)	12 (17.4%)	4 (5.8%)	30 (43.5%)	7 (10.1%)	8 (11.6%)	8 (11.6%)

ACEi: angiotensin-converting enzyme inhibitors; BB: beta-blockers; CCB: calcium channel blockers; ARB: angiotensin receptor blockers; PPL: propranolol; HCTZ: hydrochlorothiazide.

# The association ACEi + ARB (six prescriptions) was counted only once, although it was described as non-compliant in regards to ACEi and ARB.

The rate of achievement of the BP goals according to the VI DBH was 44.9%.^21^ When we considered a lower cut-off point in the case of diabetic patients (target of 130/80 mmHg), the control rate dropped to 38.6%. The BP control showed no significant association with the compliance of the prescription (Table 5). The achievement of the BP target among patients with a compliant prescription was 32.5% when the goal was BP < 140/90 mmHg and 28.6% in the case of diabetic patients with a BP goal < 130/80 mmHg.

**Table 5:** Relationship between BP control and prescription compliance according to cut-off points

	BP < 140 / 90 mmHg All patients			BP < 140 / 90 mmHg BP < 130 / 80 mmHg - diabetic patients		
	C	NC	p	C	NC	p
Prescription	N(%)	N(%)	0.75	N(%)	N(%)	0.6
Non-compliant	24 (16.1)	40 (21.9)		22 (17.2)	42 (20.6)	
Compliant	125 (83.9)	143 (78.1)		106 (82.8)	162 (79.4)	
Total	149 (44.9)	183 (55.1)		128 (38.6)	204 (61.4)	

C: controlled; NC: not controlled; p: p-value (Pearson’s chi-square test).

We conducted subgroup analyses to identify differences related to the characteristics of the population. We found no significant correlation between the compliance rate and BP control in different subgroups (elderly or sedentary individuals, women, alcoholics, smokers, blacks, and obese). A lower BP control rate was only observed in the group of elderly individuals (> 60 years), corroborating data from the literature.^25^

## Discussion

In the present study, we observed that the rate of compliance of medical prescriptions of antihypertensive drugs with the VI DBH^21^ recommendations was 80%. We found no publications in the consulted bases directly assessing the degree of compliance of prescription of antihypertensive drugs. A study in the evaluation of patterns of prescription of ACEi to users of the Brazilian Unified Health Care System (*Sistema Único de Saúde,* SUS) has shown that the average prescribed doses of captopril and enalapril maleate followed the doses recommended by the VI DBH,^21^ with only 0.3% of the patients using captopril overdoses and 0.65% using enalapril overdoses.^26^ A study on the compliance with the guidelines of the Brazilian Society of Cardiology in regards to heart failure^27^ revealed a significant gap between the practice in the primary network and the Brazilian guidelines, contrasting with what we observed in the present study in regards to hypertension.

Several studies have evaluated the most often used antihypertensive drugs in Brazil^28,29^ and demonstrated the preference for thiazide diuretics, particularly hydrochlorothiazide. Other studies have also demonstrated a preference for the prescription of ACEi in public health units.^26,30^ The findings of our study followed the same direction: the main prescribed medications by family doctors were diuretics and ACEi.

As for the number of medications used, we observed that monotherapy and dual therapy were the most frequent, which is compatible with Brazilian and international hypertension studies.^28,31^ The most frequent association of drug classes was that of diuretics with ACEi, a fact that is also in agreement with the literature.^32^ Among the non-compliances found, underdosing and underfrequencies predominated, suggesting that physicians are often slow or too cautious to intensify the antihypertensive treatment, possibly due to fear of adverse effects. We highlight the underfrequency with which captopril and nifedipine extended release were prescribed. Although the VI DBH recommend a minimum administration of twice daily, in practice many doctors are observed to prescribe these drugs only once a day.^33^

Among patients who used underdoses of negative inotropes (propranolol and diltiazem), none had a heart rate below 60 bpm, suggesting that dose escalation was an option. A possible non-compliance related to beta-blockers was the administration (especially of atenolol) to elderly individuals and patients without coronary artery disease. However, these drugs were included in the list of options for treatment of hypertension, according to the guideline in effect at the time of the data collection, and are even included in the group of drugs distributed free of charge by the government. Although these are currently known not to be antihypertensive drugs of choice, these options were considered as compliant.

We identified a lower rate of prescription of less usual medications, such as hydralazine, diltiazem, or clonidine; additionally, more than 50% of the cases of prescription errors of any kind involved these drugs. All patients using underdoses of hydralazine had systolic BP ≥ 160 mmHg and could have the doses of this medication adjusted.

We observed the non-recommended association of ACEi with ARB in 3.7% of the total ACEi prescriptions. This value corresponds to 11.5% of the total ARB prescriptions. This association is currently no longer accepted,^34^ but it was tolerated when recommended with caution in patients with proteinuria, according to the VI DBH.^21^ It should be noted that we did not evaluate the occurrence of proteinuria in the individuals evaluated in this study. However, among the patients who used ACEi associated with ARB, only one was diagnosed with chronic renal disease, corroborating the fact that the association of these medications was indeed not compliant.

Among the patients treated with three or more medications, about 90% used diuretics, showing that family doctors usually associate one diuretic in cases of multiple therapies, as recommended by the guidelines.^7,21^ With regard to the diuretics chosen, we observed that 4.8% of the patients used furosemide. Unless these individuals had some justifiable associated edematous condition (such as heart failure, chronic kidney disease, liver failure, glomerulopathy, etc.), this could have been a wrong indication. However, this was not considered a non-compliance, since this drug was part of the list of options for the treatment of hypertension, according to the VI DBH.

With respect to BP control, we found that 44.9% of the patients had BP levels within the target value < 140/90 mmHg. Obviously, there were lower rates of BP control when the target was reduced to < 130/80 mmHg in hypertensive patients with diabetes. A North-American survey^35^ has shown an estimated rate of 50.1% of BP control in 2008. On the other hand, a study in 5,023 adults in 2003 in Portugal showed that only 11.2% of the patients had BP control,^36^ while a study with more than 120,000 patients between 2005 and 2011 in Italy showed a control rate of 33.6%.^37^ In Canada, in 2009, the percentage of hypertensive patients with BP < 140/90 mmHg was 64.6%.^38^ The levels of BP control found in the present study are consistent with other Brazilian studies.^8^ A recent review has shown highly variable rates of BP knowledge (22% to 77%), treatment (11.4% to 77.5%), and control (10.1% to 35.5%), depending on the population studied.^39^ It should be pointed out that among all the cited studies of BP control, only two Brazilian studies^8^ included participants aged above 45 years, as in the present study. Knowing that the maintenance of BP < 140/90 mmHg is more difficult at more advanced ages, it can be said that in this study the frequency of BP control achieved values close to the largest values ever reported in Brazil, despite having been below 50%.

There was no statistically significant correlation between the compliance of the prescription and BP control, suggesting that the prescription, even in doses and frequencies different from those recommended by the VI DBH,^21^ was not a determinant factor in the achievement of BP goals.

Because this was a cross-sectional study, the evaluation of compliance was based on the prescription at the moment prior to the BP measurement. In other words, a prescription regarded as compliant could have the doses of the medications not yet optimized or an insufficient number of medications to achieve the desired BP goal. Also, due to the cross-sectional design of the study, the BP was measured at a single moment, which could have missed an inadequate BP control over time, or a white coat effect.^40^ Similarly, another limiting factor may have been the indication of an appropriate BP control in individuals who in fact had masked hypertension.^41^

It is worth noting that the present study evaluated the medical assistance given to hypertensive patients based on two quality aspects: the prescribed treatment and BP control. However, it is difficult to establish a direct correlation between prescription compliance and BP control, considering that a proper BP control is the result of a rather complex system involving biological, genetic, and socioeconomic factors, as well as cultural and structural sanitary aspects. We also must mention the importance of non-pharmacological measures in BP control. In this study, we did not assess non-pharmacological recommendations, which could have contributed to a better BP control. Furthermore, it can be inferred that possible non-evaluated failures in adherence or the need of a more intensive treatment, with a greater number or higher doses of medications, may be factors that contributed to a better BP control in these individuals. We did not consider the adherence to BP control because the study was aimed at analyzing the effectiveness of the therapy, and not its efficacy. Possibly, the analysis of this set of factors involved in BP control would help to find a better correlation between treatment and BP goals. On the other hand, much has evolved in recent years regarding the optimization of antihypertensive treatment, so that drugs once recommended and widely prescribed are no longer considered to be first-line choices in hypertension treatment, which is why the correlation between prescription compliance and BP control may have failed in this study.

Further studies should be performed to try to elucidate other factors involved in proper BP control, allowing the definition of quality indicators that best correlate with BP control.

## Conclusion

The degree of compliance was considered satisfactory. The rates of achievement of the recommended BP goals were compatible with those found in international studies and close to the highest values reported in Brazil. However, the achievement of the goals in 44.9% of the population in primary care, in which most of the patients are classified as having stage 1 and 2 hypertension without target organ damage, indicates that there is a long way to go and that the best investment to improve this parameter may be continuing education of physicians and other professionals involved in caring for hypertensive patients.
